# An easy-to-use pipeline to analyze amplicon-based Next Generation Sequencing results of human mitochondrial DNA from degraded samples

**DOI:** 10.1371/journal.pone.0311115

**Published:** 2024-11-21

**Authors:** Daniel R. Cuesta-Aguirre, Assumpció Malgosa, Cristina Santos

**Affiliations:** Research Group in Biological Anthropology, Biological Anthropology Unit, Department of Animal Biology, Vegetal Biology and Ecology, Universitat Autònoma de Barcelona, Bellaterra, Spain; Al Muthanna University, IRAQ

## Abstract

Genome and transcriptome examinations have become more common due to Next-Generation Sequencing (NGS), which significantly increases throughput and depth coverage while reducing costs and time. Mitochondrial DNA (mtDNA) is often the marker of choice in degraded samples from archaeological and forensic contexts, as its higher number of copies can improve the success of the experiment. Among other sequencing strategies, amplicon-based NGS techniques are currently being used to obtain enough data to be analyzed. There are some pipelines designed for the analysis of ancient mtDNA samples and others for the analysis of amplicon data. However, these pipelines pose a challenge for non-expert users and cannot often address both ancient and forensic DNA particularities and amplicon-based sequencing simultaneously. To overcome these challenges, a user-friendly bioinformatic tool was developed to analyze the non-coding region of human mtDNA from degraded samples recovered in archaeological and forensic contexts. The tool can be easily modified to fit the specifications of other amplicon-based NGS experiments. A comparative analysis between two tools, MarkDuplicates from Picard and dedup parameter from fastp, both designed for duplicate removal was conducted. Additionally, various thresholds of PMDtools, a specialized tool designed for extracting reads affected by post-mortem damage, were used. Finally, the depth coverage of each amplicon was correlated with its level of damage. The results obtained indicated that, for removing duplicates, dedup is a better tool since retains more non-repeated reads, that are removed by MarkDuplicates. On the other hand, a PMDS = 1 in PMDtools was the threshold that allowed better differentiation between present-day and ancient samples, in terms of damage, without losing too many reads in the process. These two bioinformatic tools were added to a pipeline designed to obtain both haplotype and haplogroup of mtDNA. Furthermore, the pipeline presented in the present study generates information about the quality and possible contamination of the sample. This pipeline is designed to automatize mtDNA analysis, however, particularly for ancient samples, some manual analyses may be required to fully validate results since the amplicons that used to be more easily recovered were the ones that had fewer reads with damage, indicating that special care must be taken for poor recovered samples.

## Introduction

Next-Generation Sequencing (NGS) techniques are commonly used in studies that require the sequencing of the whole genome or transcriptome, or some parts of it, in any organism, whether living or dead. NGS has many advantages over traditional capillary electrophoresis-based Sanger sequencing, such as increased throughput and depth of coverage, while reducing costs and time [1–3]. However, there are situations where the quality or quantity of the template is insufficient to obtain satisfactory results. In such cases, one solution is to use amplification-based enrichment methods. Amplicon-based NGS methods use Polymerase Chain Reaction (PCR) to increase the number of molecules for a specific region before preparing sequencing libraries. This approach can achieve higher depth coverages than common NGS techniques for a region with little off-target sequence, as the start and stop coordinates of each amplicon are predetermined [4].

The use of amplicons with NGS can greatly benefit the analysis of human mitochondrial DNA (mtDNA) non-coding region (NCR) in both forensic and population genetics. The NCR is a region of almost 1120 base pairs (bp) that exhibits high levels of variation in human populations, especially in the two hypervariable regions HVRI (16024–16383 bp) and HVRII (57–372 bp). Traditionally, these two hypervariable regions were amplified by PCR and sequenced using Sanger technology [5]. Nonetheless, amplicons with over 300 bp may not be reliable in samples of poor quality, so shorter amplicons should be used instead. This approach requires many PCR amplification reactions and Sanger sequencing reactions, which can become very time-consuming and demanding [6].

As previously mentioned, NGS technology provides a larger amount of genetic information more efficiently than electrophoresis-based methods [7, 8]. As a result, some commercial kits that allow mtDNA-NCR analysis using NGS have been developed. In the specialized field of forensic genetics, three distinct kits have been developed: The ForenSeq mtDNA Control region kit (Verogen), the Precision ID mtDNA control region Panel Kit (Thermo Fisher Scientific), and the PowerSeq^TM^ CRM Nested System kit (Promega Corporation). Both the ForenSeq mtDNA Control Region kit (Verogen) and the Precision ID mtDNA control region Panel Kit (ThermoFisher Scientific) require two PCR reactions to amplify the entire mtDNA-NCR. Additionally, another PCR step is needed to add adapters and index the samples. In contrast, the PowerSeq^TM^ CRM Nested System kit (Promega Corporation) streamlines the process by using a single-multiplex PCR reaction to both amplify the mtDNA-NCR and add indexed adapters for Illumina MiSeq^TM^ (Illumina, INC., San Diego, CA, USA). This approach significantly reduces overall work time, minimizes amplicon manipulation, and lowers the risk of contamination. Traditionally, the sequencing data obtained from these three kits has been analyzed with the MiSeq FGx and UAS software (Verogen), the Torrent Variant Caller software (ThermoFisher Scientific), and the GeneMarker® HTS software (Promega Corporation), respectively [6, 9–17]. However, these software packages lack specific tools for dealing with particularities of genetic data derived from ancient and forensic samples, that are presented afterward.

Ancient DNA (aDNA) refers to the study of DNA extracted from preserved or fossilized biological material of specimens that perished long ago [18]. It is essential to keep in mind that these samples undergo post-mortem DNA damage due to environmental conditions and degradation reactions. For instance, they may experience extensive fragmentation, abasic sites, single-strand breaks, interstrand cross-links, and some chemical modifications of nucleotide bases [19–22].

Post-mortem molecular damage (PMD) in DNA can hinder the study of aDNA as undamaged DNA molecules can outcompete ancient templates in enzymatic reactions [23]. To authenticate aDNA, researchers often analyze sequence length and specific nucleotide misincorporation patterns. These patterns include both the short length of the sequences and an excess of cytosine to thymine (C-to-T) misincorporations at 5’ ends and its complementary guanine to adenine (G-to-A) misincorporations at 3’ ends [24]. Various tools are available for detecting patterns of misincorporation occurring at the ends of molecules. Among these tools are MapDamage2 [25] and Shmutzi [26], which can help to estimate contamination levels and authenticate aDNA by using these misincorporations at the fragment ends. Additionally, PMDtools is a bioinformatic tool that uses the PMD score (PMDS), a log-likelihood ratio, to analyze damage in sequencing data. When the PMDS is negative, PMDtools identifies and selects reads affected by PMD, PCR errors, and sequencing misreads. On the other hand, when the PMDS is positive, it focuses only on changes caused by DNA degradation. The PMDS value indicates the likelihood that a change is due to DNA degradation, with higher values implying fewer available reads. Skoglund et al. demonstrated the effectiveness of PMDS in analyzing ancient samples from different periods. For example, samples over 38,000 years old and around 5,000 years old were well-suited to a PMDS higher than 2. However, for more recent samples, approximately 100 years old, a high PMDS value removed almost all reads and lower values of PMDS must be used [27].

When employing amplicon-based techniques for sequencing analysis of damaged DNA, there is a replication of the original molecules, leading to a surplus of identical sequences that can overshadow some polymorphic variants. Hence, it is essential to remove duplicates in all NGS analyses that involves ancient or degraded samples [28]. For present-day samples, it has been stated that deduplication has a minimal effect on the accuracy of variant calls, so it is not necessary to carry it out [29]. Duplicate reads encompass PCR duplicates (reads derived from the same original fragment in the DNA library) or optical duplicates (reads from clusters read twice in Illumina platforms). There are available numerous tools to eliminate duplicate reads. Samtools’ ‘markdup’ command [30], DeDup software [31], and Picard’s ‘MarkDuplicates’ command [32] operate by eliminating duplicates according to template coordinates. Conversely, the ‘dedup’ parameter from fastp [33] identifies duplication only when all base pairs match between reads. This functionality was introduced in fastp v0.22.0.

There are pipelines available to analyze aDNA data generated by NGS technologies, such as the nf-core/eager pipeline [34]. However, the nf-core/eager pipeline (v2.5.0) does not yet have a tool to eliminate duplicates based on reads with identical coordinates. Specific pipelines to analyze ancient mtDNA data obtained by NGS are also available, such as the pipeline presented by Diroma et al. [35], but it is not designed to analyze amplicon-based data. As far as we know, no free software is available to analyze data from ancient and forensic mtDNA-NCR specifically generated by amplicon-based NGS methods. Therefore, our main goal is to develop a free and easy-to-use bioinformatic tool to analyze data from ancient or degraded samples obtained by an amplicon-based Next Generation Sequencing method. Also, we will determine the best deduplication method to work with amplicons and establish the best PMDS from PMDtools that can differentiate mtDNA-NCR from present-day and ancient samples.

## Material and methods

### Samples

Following the processing of numerous samples in our laboratory, 148 high-quality ancient Spanish human samples were carefully selected, encompassing various chronologies. No permits were required for the described study, which complied with all relevant regulations. Moreover, 15 samples from members of the research group (all members provided confirmed consent), which were used as contaminant control, were also selected. All samples exhibited a recovery rate of over 80% for the mtDNA-NCR, and none displayed mixed bases exceeding the designated threshold of 30% of the reads.

### Sample preparation and DNA extraction

The external surface of all skeletal remains from ancient samples was polished using a sterile tungsten tip placed into a micro drill to remove the outermost millimeters. Then, a 2–3 cm section was cut using a tungsten sterile disk and pulverized with a hammer. After that, 100–150 mg of sample was used for DNA extraction.

DNA was extracted in the ancient DNA Laboratory of the Biological Anthropology Unit of the Universitat Autònoma de Barcelona (UAB), Spain, using the High Pure Viral Nucleic Acid Large Volume kit (Roche), which is a silica-based method on a HE-membrane. The extraction was carried out following the specifications of Vinueza-Espinosa et al. [36]. Blank controls were processed with every extraction to ensure accuracy.

For present-day individuals, buccal swab samples were taken. DNA was extracted using the QIAamp DNA Investigator Kit (Qiagen) in the pre-PCR area of the modern DNA Laboratory of the Biological Anthropology Unit of the UAB.

### Amplification, libraries preparation, and sequencing

All the samples were amplified and indexed using the PowerSeq^TM^ CRM Nested System kit (Promega Corporation). The NCR was amplified from base 16013 to base 592, with 10 overlapped amplicons, as follows: Amplicon 1 (16013–16126 bp), Amplicon 2 (16116–16225 bp), Amplicon 3 (16223–16408 bp), Amplicon 4 (16387–16486 bp), Amplicon 5 (16474–30), Amplicon 6 (16555–152 bp), Amplicon 7 (136–257 bp), Amplicon 8 (246–364 bp), Amplicon 9 (342–436 bp), and Amplicon 10 (429–592 bp).

For the ancient samples, the modified protocol proposed by Vinueza-Espinosa et al. [37] for archaeological samples was used. The PCR conditions were as follows: 96°C for 15 min; 35 cycles of 96°C for 15 seconds, 60°C for 35 seconds and 72°C for 30 seconds; and 60°C for 2 min. For present-day samples, the standard conditions proposed by the manufacturer were followed.

After amplification/indexation, the libraries were purified using the GeneRead^TM^ Size Selection kit (Qiagen), following the manufacturer’s protocol. Quantifications of the libraries were performed with Qubit^TM^ ds HS Assay kit (ThermoFisher Scientific), and the libraries’ concentrations were normalized to 4 nM. Runs were performed on the Illumina MiSeq instrument, using standard flow cells, MiSeq® Reagent kit v2 Nano 2x150bp.

### Bioinformatic tool selection for duplicate read identification

**Fig 1:**
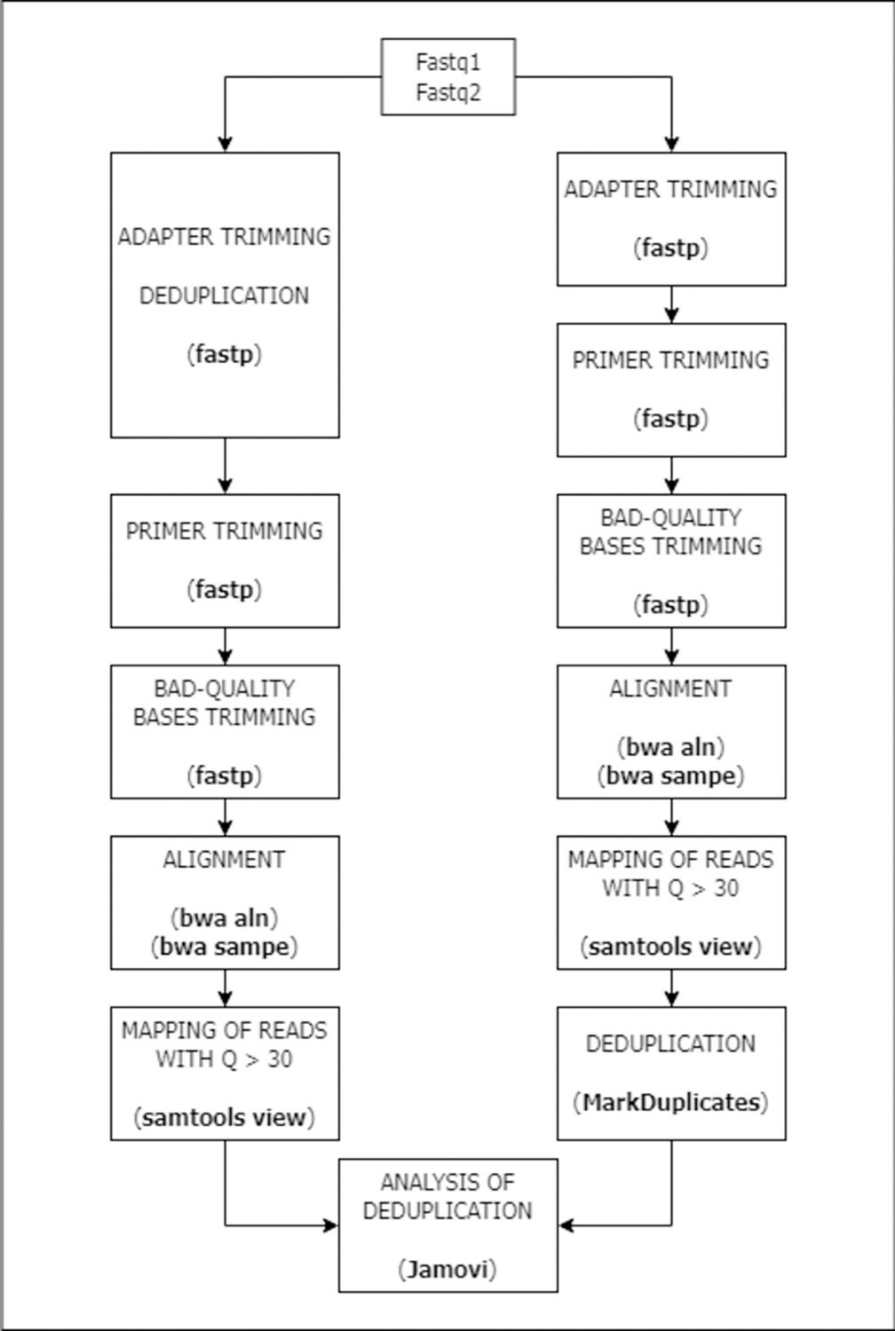
Workflow followed to study what method to remove duplicates brings better results.

Two similar workflows, each with the same filters, were created to compare which deduplication (identification and elimination of duplicates) method was the best to remove duplicates correctly from ancient amplicon-based NGS sequences. Two tools based on different duplicates detection were compared. On the one hand, fastp v0.23.2 [33] was used, which is a tool that identifies duplicates when two sequences are identical, meaning that they have the same sequence throughout the whole molecule. On the other hand, ‘MadkDuplicates’ from Picard v2.26.2 [32], was employed as a proxy of those tools that remove duplicates according to the template’s coordinates. The deduplication tool was placed for each workflow as shown in Fig 1. For one workflow, deduplication was performed using fastp v0.23.2 [33] after adapting trimming (the process of adapters’ elimination). For the other workflow, deduplication was performed using ‘MadkDuplicates’ from Picard v2.26.2 [32] after selecting reads mapped with a quality score over 30. The number of useful reads, defined as the reads that passed all filters and allowed for variant calling, and the duplication rate, which was calculated as one minus the number of reads without duplicates divided by the number of reads with duplicates (equivalent to the percentage of duplicates), were both annotated.

The Integrative Genome Viewer (IGV) v2.11.2 [38] was employed to analyze which tool removed more true duplicates and retained more non-duplicated sequences.

To compare the number of useful reads and the duplication rate between different deduplication methods normality was checked using the Shapiro-Wilk test and the differences between the deduplication methods were studied using a Paired Samples T-Test.

### PMDS selection for aDNA authentication

To determine if ancient samples can be authenticated—distinguishing them from present-day samples—based on their percentage of reads with PMD, five PMD thresholds (PMDS: -1, 0, 1, 2, and 3) were used in PMDtools 0.23.2 [27], and the percentage of reads with PMD identified for each threshold. An initial exploratory analysis was conducted, and the 95% Confidence Intervals for each percentage were estimated. To assess the variations in the percentage of reads with PMD between chronological age and PMDS thresholds, a Repeated Measures ANOVA test was performed, along with their respective Post Hoc Comparisons, employing Bonferroni’s correction. Ancient samples were deduplicated prior PMDS selection analysis, while present-day samples were not.

### Study of differences by amplicon

The depth coverage and percentage of damage for each amplicon were estimated, including Amplicon 1 (16013–16126 bp), Amplicon 2 (16116–16225 bp), Amplicon 3 (16223–16408 bp), Amplicon 4 (16387–16486 bp), Amplicon 5 (16474–30), Amplicon 6 (16555–152 bp), Amplicon 7 (136–257 bp), Amplicon 8 (246–364 bp), Amplicon 9 (342–436 bp), and Amplicon 10 (429–592 bp). A PMDS of 1 was used for both ancient and present-day samples to determine the level of damage in each amplicon. The mean and median of depth coverage and percentage of damage were computed. Furthermore, for both ancient and present-day samples, a correlation between the depth coverage and the percentage of damage in amplicons was performed.

All the statistical analyses were carried out using Jamovi 2.3 [39, 40] and considered a 5% significance.

## Results

### Selection of bioinformatic tools to remove duplicated reads and thresholds of PMDtools to authenticate aDNA

After applying the established filters, the analysis of deduplication and PMD was performed including 148 ancient samples and 15 present-day samples (S1 File).

Upon conducting an exploratory analysis of both the number of useful reads and the duplication rate of the data a few values were identified as outliers. However, they were considered acceptable after thorough analysis. Consequently, all the data was used in subsequent analyses.

When the samples were analyzed with fastp v0.23.2, there were a higher number of useful reads and a lower duplication rate compared to the results obtained with MarkDuplicates (Table 1), being the differences between the two methods significant (Wilcoxon: p < 0.001).

**Table 1:** Comparative descriptive statistics of useful reads count and duplication rates across the two deduplication strategies.

	Deduplication method	Mean	Median	IQR	Percentile 2.5^th^	Percentile 97.5^th^	Minimum	Maximum	Sapiro-Wilk	
									W	p
**Number of useful reads**	**fastp**	18,932.5	17,108	15,696.5	3,626.5	42,425.5	1950	47226	0.96	< 0.001
	**MarkDuplicates**	107.6	102	50	45.75	207.0	28	225	0.96	< 0.001
**Duplication rate**	**fastp**	28.5	29.8	20.63	2.75	60.3	0.48	67.7	0.98	0.010
	**MarkDuplicates**	99.6	99.7	0.15	99.1	99.8	98.63	99.9	0.76	< 0.001

By visualization of deduplicated samples with IGV v2.11.2, one the one hand, it was seen that MarkDuplicates removed sequences due to the identical coordinates, despite the differences at the sequence of the different reads (S1 Fig). Meaning that MarkDuplicates remove non-duplicate sequences. On the other hand, fastp toke sequences into account and allowed to retain more original molecules.

Table 2 presents descriptive statistics for the percentage of recovered reads with damage in ancient and present-day samples when different PMDS values were considered. It is evidenced that the higher the PMDS, the lower the percentages of recovered reads. In accordance with what is expected for the relatively recent chronology of the samples (some samples classified as ancient belong to the 20^th^ century), values of PMDS of 2 or 3 identify a very low percentage of reads with damage, limiting subsequent analysis based only on damaged reads. However, for PMDS 1, the percentage is higher, with a median value reaching 25% for ancient samples, and of important relevance, clearly different between present-day and ancient samples.

**Table 2:** Descriptive statistics of the percentage of recovered reads with damage when different post-mortem molecular damage scores (PMDS) were used in present-day and ancient samples.

	PMDS in PMDtools	Chronology	Mean	Median	IQR	Minimum	Maximum	Percentile 2.5^th^	Percentile 97.5^th^
**Percentage of recovered reads with damage**	**-1**	**Present-day**	74.91	75.76	8.15	67.30	83.18	75.33	85.08
		**Ancient**	94.92	95.23	5.67	86.62	99.87	87.40	99.59
	**0**	**Present-day**	8.74	10.51	9.73	2.18	15.76	2.28	15.12
		**Ancient**	32.41	31.79	15.15	8.76	67.45	13.82	56.35
	**1**	**Present-day**	1.65	1.35	1.31	0.78	2.89	0.82	2.84
		**Ancient**	26.57	25.86	12.59	7.49	62.92	9.17	47.74
	**2**	**Present-day**	0.56	0.31	0.57	0.18	1.35	0.19	1.26
		**Ancient**	12.28	11.53	8.06	2.01	31.53	3.33	23.32
	**3**	**Present-day**	0.33	0.11	0.38	0.08	1.14	0.08	1.06
		**Ancient**	7.44	7.01	5.36	1.06	19.02	1.69	15.74

**Fig 2:**
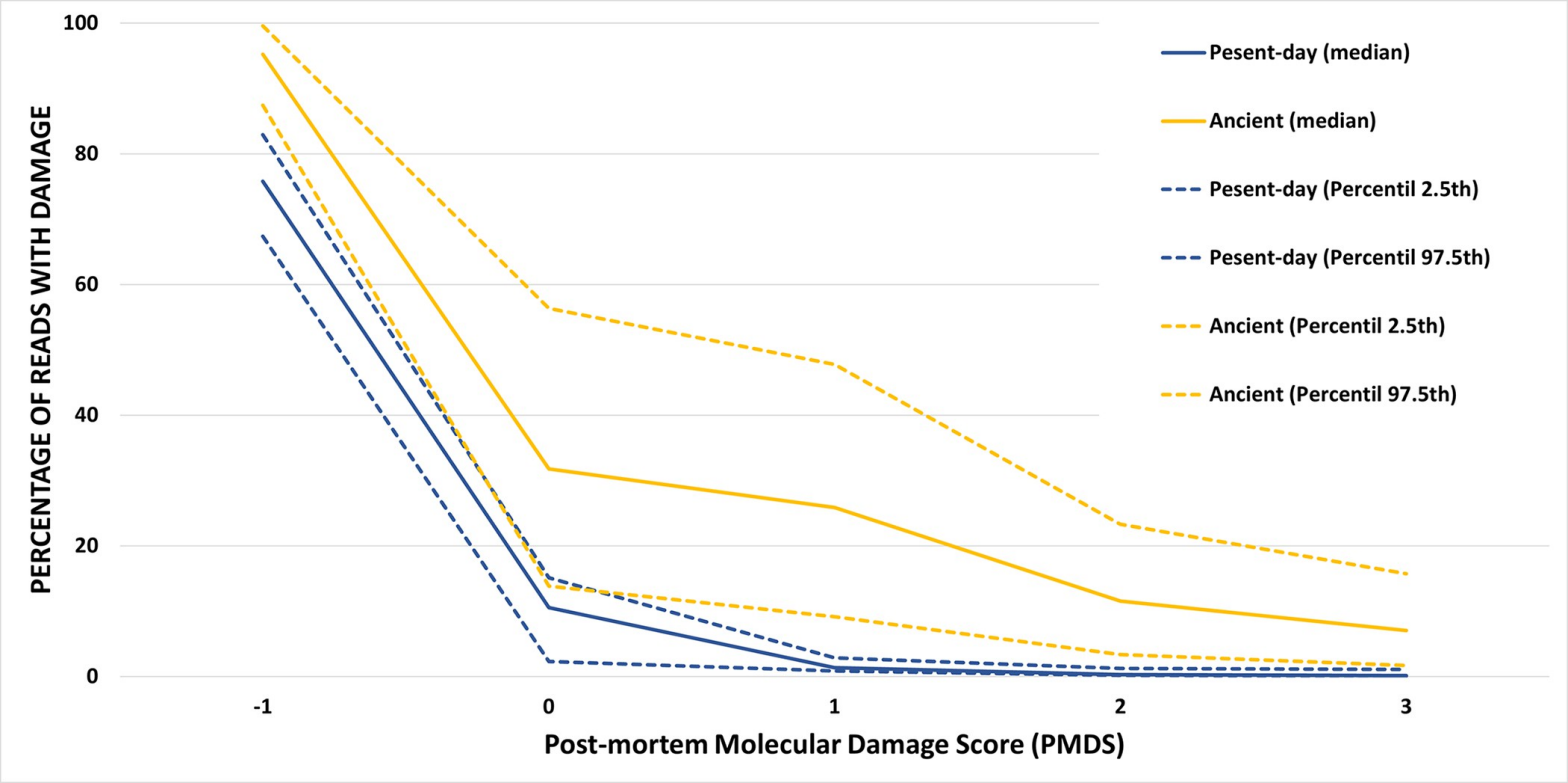
Percentage of damage with different thresholds from PMDtools. The percentage of reads with post-mortem molecular damage (PMD) extracted by PMDtools (Y axis) with five different PMD scores (PMDS) (X axis). These percentages have been estimated by dividing the number of reads with damage given by PMD (reads with post-mortem molecular damage) by the number of reads aligned with the reference sequence. Medians, 2.5^th^, and 97.5^th^ percentiles are indicated for ancient and present-day samples. For both ancient and Present-day samples, the higher the PMDS the lower the percentage of reads recovered with damage. Ancient reads presented higher percentages of damage than Present-day samples.

When the chronology was considered, in each PMDS analyzed, ancient samples had a higher percentage of reads with damage than present-day samples. The 2.5^th^ and 97.5^th^ percentiles between present-day and ancient samples overlapped only for PMDS = 0 (Table 2 and Fig 2).

The significance of differences in the percentage of recovered reads with PMD was confirmed for both chronology (Repeated Measures ANOVA: F = 127; p-value < 0.001) and PMDS (Repeated Measures ANOVA: F = 2291.4; p-value < 0.001). Moreover, pairwise comparisons for the chronology in each PMDS also showed significant differences (p-value < 0.001).

### Differences by amplicon

**Fig 3:**
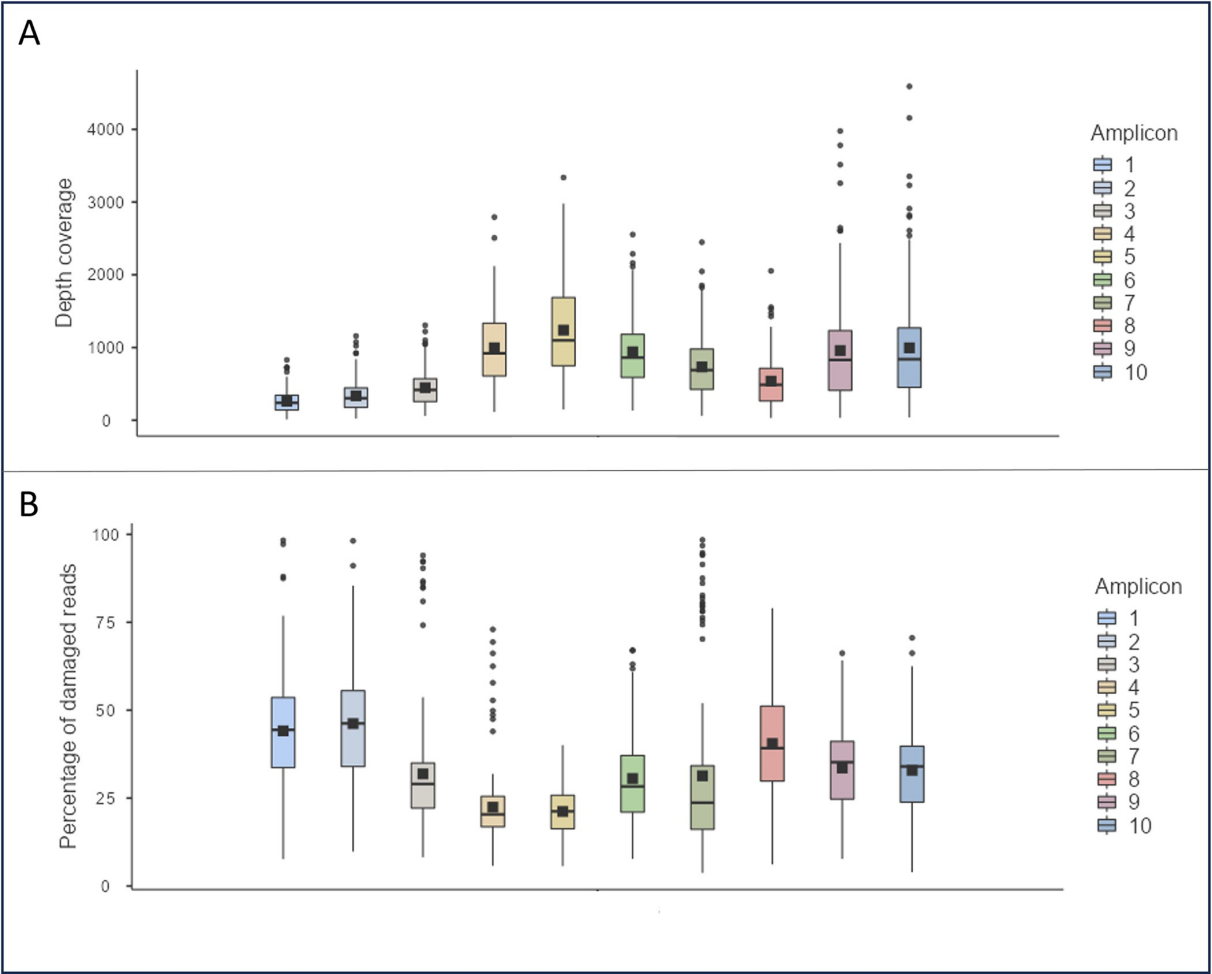
Comparison between the 10 amplicons amplified with the PowerSeq^TM^ CRM Nested System kit (Promega Corporation) in ancient samples. Box-plot of (A) the depth coverage (number of unique reads aligned with that amplicon) and (B) the percentage of damaged reads (percentage of reads that presented post-mortem molecular damage, according to PMDtools). They are estimated from the 148 ancient samples in each one of the ten amplicons amplified with the PowerSeq^TM^ CRM Nested System kit (Promega Corporation) (marked with different colors and sorted from 1 to 10). Points represent outliers and square marks mean. The higher the depth coverage, the lower the percentage of damaged reads and vice-versa.

**Fig 4:**
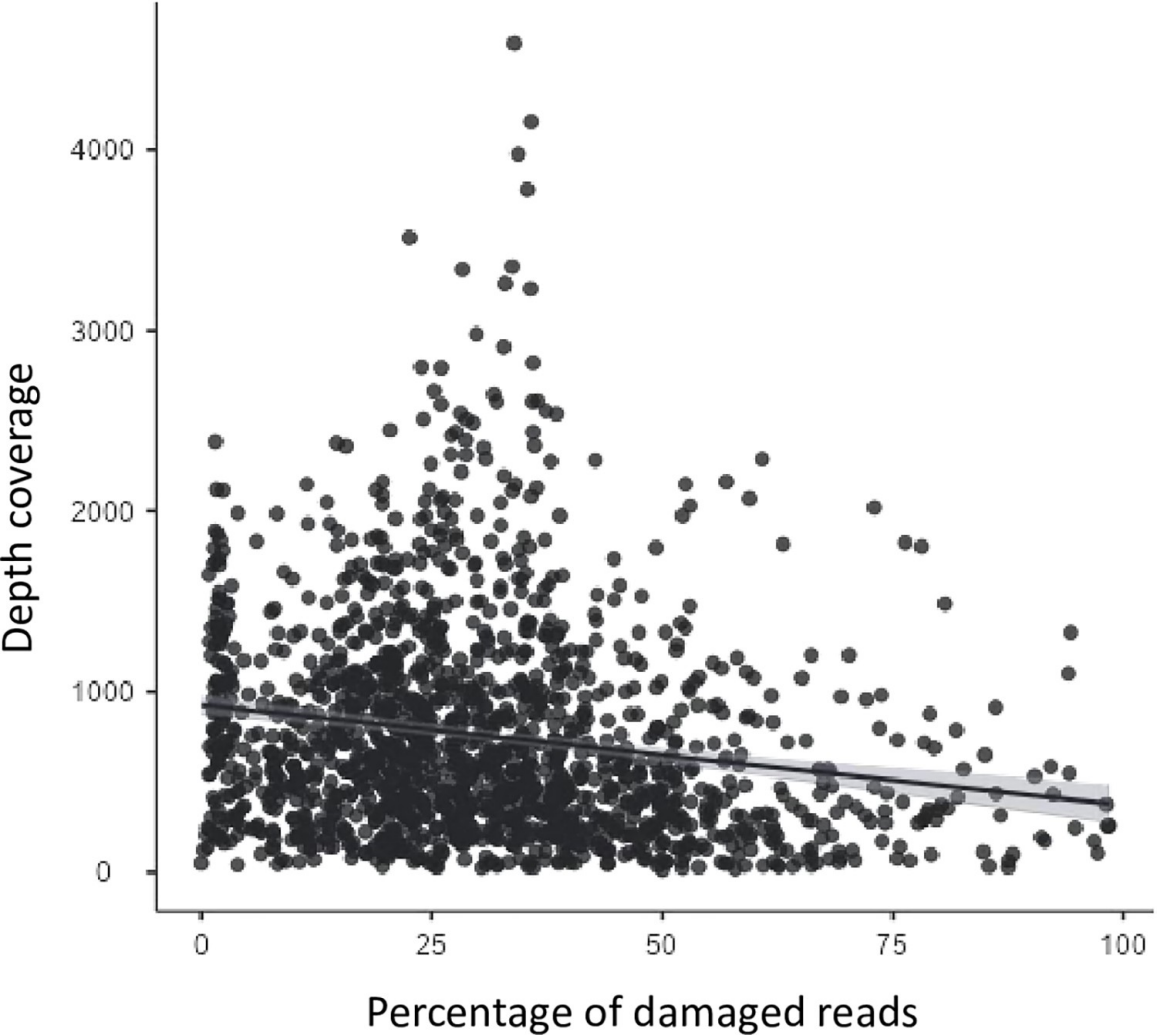
Relation between depth coverage and percentage of damage. Scatterplot that relates the depth coverage (Y axis) against the percentage of reads that presented post-mortem molecular damage when a PMDS of 1 was used in PMDtools (Y axis) for all samples and all the regions of the non-coding region of the mtDNA.

Considering the parameters previously validated (fastp to eliminate duplicated reads and PMDS = 1), the results for depth coverage and the percentage of damaged reads for each amplicon were calculated and are available in S1 File for both present-day and ancient samples. The median and mean values, for ancient samples, are shown in Table 3 and Fig 3. In ancient samples, amplicons with the lowest depth coverage have the highest damage values (Amplicon 1: median depth coverage = 235.75, median percentage of damage = 44.41; Amplicon 2: median depth coverage = 301.60, median percentage of damage = 46.10). In contrast, amplicons with the highest amount of reads have the lowest levels of damage (Amplicon 4: median depth coverage = 911.55, median percentage of damage = 20.29; Amplicon 5: median depth coverage = 1074.21, median percentage of damage = 21.17). For present-day samples, the relationship between depth coverage and the percentage of damaged reads was less clear when analyzing the descriptives. However, for both ancient and present-day samples together there is a significant negative correlation between the percentage of damaged reads and the median depth coverage (Sperman’s rho = -0.187, p-value < 0.001), as it can be seen in Fig 4; as well as when ancient and present-day samples were analyzed separately (ancient Spearman’s rho = -0.174, p-value < 0.001; present-day Spearman’s rho = -0.224, p-value = 0.006).

**Table 3:** Mean and median depth coverage and damage percentage (PMDS = 1) per amplicon for ancient samples.

Amplicon	Depth coverage: mean (SD)	Percentage of damage: mean (SD)	Depth coverage: median (IQR)	Percentage of damage: median (IQR)
**1**	257.86 (168.15)	43.89 (16.67)	235.75 (212.60)	44.41 (20.29)
**2**	332.24 (228.92)	45.78 (18.41)	301.60 (265.84)	46.10 (21.75)
**3**	443.70 (254.27)	31.54 (17.34)	413.12 (318.19)	28.90 (13.03)
**4**	985.39 (532.46)	22.18 (11.51)	911.55 (736.21)	20.29 (8.55)
**5**	1225.41 (651.26)	20.97 (7.33)	1074.21 (941.31)	21.17 (9.77)
**6**	931.64 (501.42)	30.23 (14.05)	853.22 (601.34)	28.15 (15.75)
**7**	725.81 (454.60)	30.96 (23.34)	671.35 (554.41)	23.42 (17.96)
**8**	531.57 (368.32)	40.11 (16.61)	479.47 (467.24)	39.03 (22.28)
**9**	948.08 (759.52)	33.19 (13.44)	821.32 (832.29)	34.96 (17.20)
**10**	984.11 (810.61)	32.48 (13.39)	817.35 (809.48)	33.87 (15.85)

### Pipeline development

**Fig 5:**
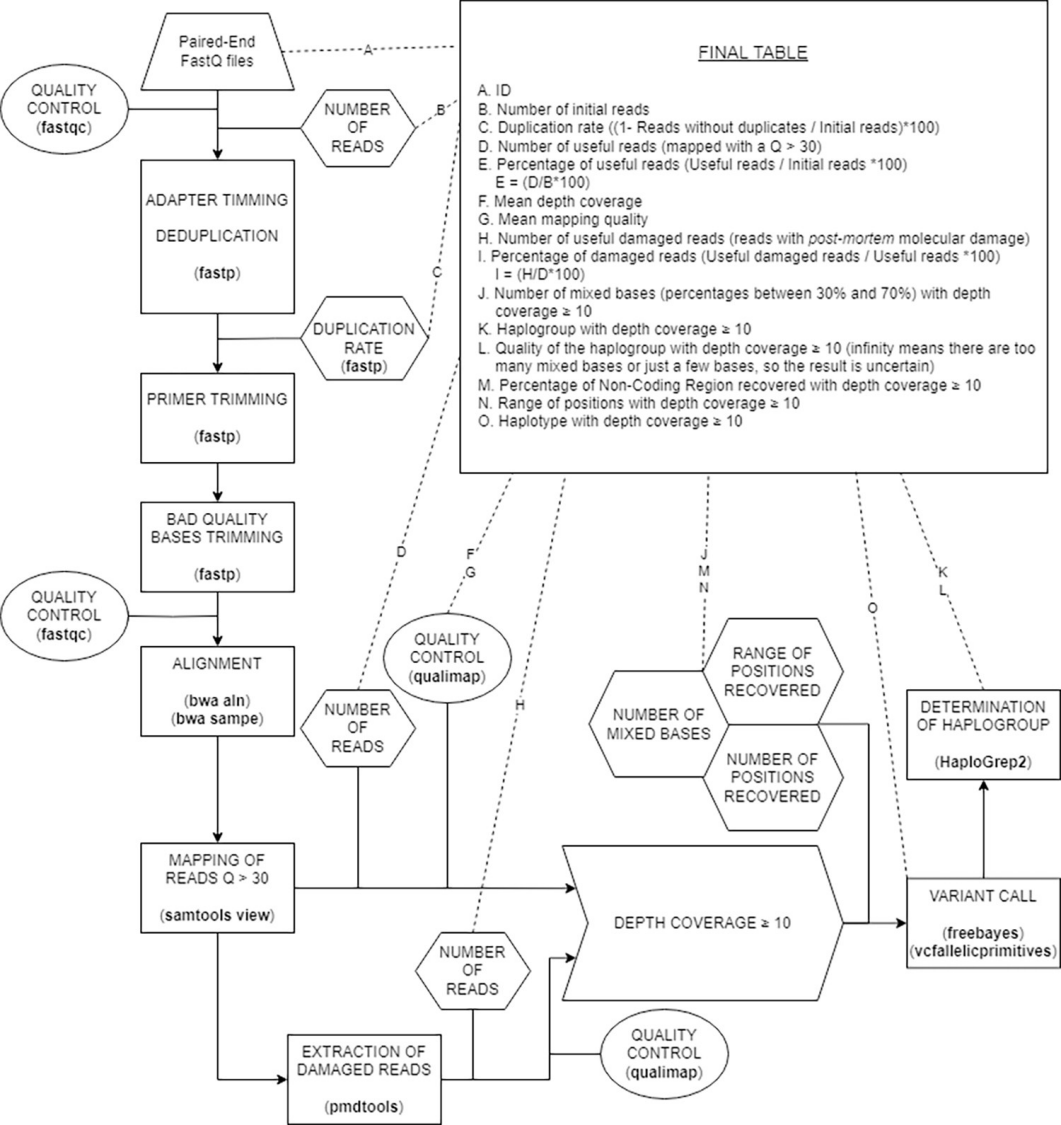
Pipeline followed to generate results from paired-end reads obtained by amplicon-based NGS methods. Tools are marked in bold while the processes are in capital letters. The minimum depth coverage can be easily changed at the top of the script.

Deduplication using fastp and a threshold of 1 in PMDtools were included in an aDNA pipeline to analyze the NCR of mtDNA reads generated from PCR-based libraries (Fig 5).

The preprocessing of the reads involved quality-check analysis and processing of both 5’ and 3’ ends of each read to obtain better quality mapped reads. Raw paired-end FastQ files were first subjected to quality check using FastQC v0.11.9 [41]. Duplicates, adapters, primers, and bad quality bases in 3’ ends were eliminated by three consecutively fastp v0.23.2 [33] analyses. Initially, a fastp v0.23.2 [33] analysis was performed to trim the adapters and remove the duplicates, restricting the minimum length of the reads to 30 bp and trimming the poliX of the tails. Another fastp v0.23.2 [33] analysis was used to eliminate primers. Since these sequences are unknown and unidentified by fastp v0.23.2, 25 bp from the 5’ end and 5 bp from the 3’ end were trimmed for both paired-end reads. Finally, the last fastp v0.23.2 [33] was used to eliminate bases with a quality lower than 20 from the 3’ end, if any. Additionally, in this last analysis, overlapped paired-end reads were merged if the overlapping region was higher than 10 bp. Another FastQC v0.11.9 [41] was used to check the quality of the trimmed FastQ files.

The reads were aligned to the non-coding region of the revised Cambridge Reference Sequence (rCRS, GenBank accession NC_012920.1) [42]. This alignment was done using BWA v0.7.17 [43]. To avoid issues caused by the circularity of the mtDNA and the reads aligned in the replication origin, the sequence was linearized from base 15901 to base 700. Later, position renumbering was made as position 15901 from the mtDNA was deemed position 1 in the new reference sequence.

To generate a sorted BAM file that only contained useful reads, SAMtools v1.16.1 [30] was used, and reads with a mapping quality of over 30 were included. Then, the samtools view -c command was applied to estimate the number of useful reads, those that would be used to determine the haplotype and haplogroup. Next, reads that presented PMD were extracted using PMDtools v0.60 [27] and saved in a separate BAM file. The number of reads with PMD was then estimated using the samtools view -c command.

QualiMap v2.2.2a [44] was used to quality-checking of BAM files, allowing both mean depth coverage (*mean coverageData* in the output of QualiMap) and mean mapping quality (*mean mapping quality* in the output of QualiMap) to be extracted by command *grep*.

From this point forward, the same procedures were applied to both BAM files containing all reads and BAM files containing only those reads exhibiting *post-mortem* molecular damage that were extracted by PMDtools. The range of positions with more than 10 reads was determined using the ‘samtools depth’ command and a Python script. Variant calling was performed with FreeBayes v1.3.6 [45] and vcfallelicprimitives from VCFlib v1.0.3 [46]. In this analysis, the minimum allele depth was set at 10 reads (parameter: min-coverage = 10) and the threshold for minor variant frequency call was established at 30% (parameter: F = 0.30). Indels, multi-nucleotide polymorphisms, and complex events were excluded from the analysis, as well as the region between bases 303 and 315 due to their alignment complexity. Positions with more than one nucleotide variant with a frequency ranging between 30% and 70% were classified as mixed bases, and each instance was recorded for further examination. In such cases, the International Union of Pure and Applied Chemistry (IUPAC) nomenclature was employed in variant call.

An HSD file was generated, containing the sample name, the range of the mitogenome covered by more than 10 reads, an interrogation mark (representing the unknown haplogroup), and the haplotype; all these were separated by tabs. Finally, Haplogrep2 v2.4.0 [47] was employed locally to predict mtDNA haplogroups.

Several outputs are provided (summarized in S1 File). The first output is a table that includes the ID, number of initial reads, duplication rate, number of useful reads, percentage of useful reads, mean depth coverage, mean mapping quality, number of useful damaged reads, percentage of damaged reads, and number of mixed bases. It also includes the most probable haplogroup, quality of that haplogroup, range of positions, and haplotype for a depth coverage ≥ 10. Secondly, an HSD file is generated for uploading to Haplogrep3, offering more specific information not available from the local analysis. This file contains all pertinent details for the two types of BAM files, including those with all reads and those composed only by the reads with post-mortem molecular damage extracted by PMDtools for depth coverage ≥ 10. Finally, a TXT file containing information on the mixed bases is provided. This information includes the sample ID, position, alternative base, depth coverage of the position, number of appearances of the alternative base, and percentage of the alternative base. A mixed base will only be reported if the percentage of the mixture falls between 30% and 70%.

Some parameters, such as the reference sequence, the minimum allele depth, and the minor variant frequency can be changed at the top of the script.

The pipeline and its usage description are available at https://github.com/DanielRCA/NCR-mtDNA_ampliconbasedngs.

## Discussion

Amplicon-based NGS methods offer the advantage of yielding a high number of reads from regions with few template sequences. However, this process generates a significant number of duplicated reads, which must be eliminated to analyze just unique reads. There are numerous tools for removing duplicates. Some are based on query or template coordinates (the positions where reads start and/or finish), such as the ‘markdup’ command from SAMtools [30], DeDup software [31], and ‘MarkDuplicates’ from Picard [32]. Nonetheless, since amplicons are reads with identical coordinates, these tools may not be suitable. For this purpose, the ‘dedup’ parameter in fastp, implemented in v0.22.0, should be the best option because it identifies duplicates only when all base pairs between reads are identical [33]. The number and percentage of reads remaining after duplicate elimination were analyzed using two different tools: MarkDuplicates and fastp. Using MarkDuplicates, over 99% of reads were removed. In contrast, fastp removed an average of 30% of the reads as duplicates. These differences were significant, indicating that fastp removes fewer duplicates in amplicon-based studies, as we expected for the characteristics of each tool. Although both tools retained some true duplicates, MarkDuplicates also removed some molecules with the same coordinates and differences in sequence, as showed with the manual analysis with Integrative Genome Viewer (IGV) v2.11.2 [38]. Overall, it is evidenced that making the deduplication with fastp is a better approach when amplicons derived from degraded or damaged samples are involved.

The analysis of ancient or degraded samples presents the issue of *post-mortem* molecular damage (PMD) misincorporations, specifically C-to-T at 5’ ends and their complementary G-to-A misincorporations at 3’ ends [24]. However, when using amplicons, misincorporations can occur throughout the entire sequence. This is because amplicons may not extend to the molecule ends due to their predetermined coordinates. Consequently, tools like MapDamage2 [25] or Schmutzi [26] become less effective for amplicon analysis, as they primarily detect misincorporation patterns at the sequence ends, making this kind of sample appear as if they were not ancient samples. Unlike these tools, PMDtools does not limit DNA damage patterns to the sequence ends [27], calculating the ratio of reads with damage, a viable method for authenticating ancient DNA (aDNA) sequenced by NGS from amplicons.

The variation of the percentage of PMD in the NCR of mtDNA between ancient and present-day samples was investigated by using different Post-mortem Molecular Damage Scores (PMDS) to establish a threshold for distinguishing ancient from present-day samples. This approach could also aid in identifying contaminations. A PMDS of -1, which ignores any damage, was used as control. Skoglund et al. [27] already stated that high thresholds of PMDS would work great for really ancient samples (> 5,000 years old), but it would remove almost all sequences for more recent ancient samples (100 years old). As both recent ancient samples and more ancient samples were analyzed, only a PMDS of 1 or 2 should be used. Considering that higher PMDS results in fewer reads recovered [27], we opted for a PMDS of 1 to ensure enough reads for downstream analysis. With this PMDS, we determined that an ancient sample with 80% of the NCR recovered could be considered ancient with a 95% confidence if the percentage of damage is higher than 9.17%. For lower values, caution is advised, due to possible present-day contamination.

Some authors have described that PMD is not randomly distributed [48–51], so the frequency of damage could vary depending on the region being analyzed. Therefore, differences between amplicons must be considered. This is particularly important for samples for which not all amplicons are recovered, as the value obtained with PMDtools could be biased. It can be observed that amplicons with the highest depth coverage (amplicons 4 and 5) tend to accumulate shorter reads (20–40 bp) with less damage. Additionally, a negative correlation between depth coverage and the percentage of damage in both ancient samples and present-day samples was found. These findings could be explained by the possibility that damage accumulates at the binding sites of some primers, potentially acting as a hotspot of damage and hindering primer binding [37, 48–51]. The results in present-day samples are nearly non-significant, which could be due to the low amount of damage typically found in these samples [49, 51–54].

It is important to consider that for samples with poor quality–for example, those that only amplify two amplicons (4 and 5)–the percentage of damage is likely to be underestimated and may fall below the threshold we have presented in this study for ancient samples (9.17%). Nonetheless, we offer another method to detect potential contamination, which is also useful in cross-contamination: the examination of mixed bases. When mixed bases appear in a low-coverage mitochondrial genome, several factors could be the cause: *post-mortem* molecular damage, heteroplasmy, or contamination. Working with amplicons, depth coverages tend to be high, so it would be difficult for PMD to be a source of mixed bases [37]. Heteroplasmy–a condition in which an individual has different types of mtDNA–is a natural phenomenon that varies among individuals from the same and different populations and, even within tissues from the same individual [55–57]. The only study that used bones for the analysis of the NCR identified that 19% of their sample (17 out of 87 individuals) exhibited at least one heteroplasmy: fourteen individuals had one heteroplasmy, two individuals had 2 heteroplasmies, and one individual had three heteroplasmies [55]. In samples where more than one mixed base is observed, a more detailed study should be conducted, as it is quite likely to indicate contamination. Haplotypes that are generated exclusively from BAM files containing only the damaged reads can help to identify the presence of contamination since these BAM files include only ancient reads. By comparing both the haplogroup and haplotype of BAM files that include only damaged reads with the results obtained for the BAM file with all the reads, it is possible to determine the presence of contamination. Moreover, using the IGV program [38] to visualize the alignments can improve the analysis of regions that contain multiple mixed bases, as particular patterns can be observed, and multiple mitochondrial profiles may be detected.

Using all the previously mentioned considerations we propose the implementation of a new pipeline that consists of a group of tools designed to extract information about the mtDNA-NCR for ancient or degraded samples that have been sequenced using an amplicon-based NGS method. Virtually the tool can be applied to mtDNA-NCR data generated with different commercial kits or other custom amplicon panels. The pipeline produces a final table that includes the sample identifier, total number of reads obtained from sequencing, number of deduplicated reads, duplication rate, number of useful reads, percentage of useful reads, mean depth coverage, number of reads with PMD, percentage of reads with PMD, number of mixed bases, haplogroup, quality of the haplogroup according to Haplogrep2 [47], percentage of NCR recovered, range of positions with coverage over 10 reads, and haplotype. Additionally, an HSD file with the haplotypes for both BAM files with useful reads and BAM files with only reads with PMD is also generated. Finally, a description of the mixed bases is also provided in a different TXT file.

The circular nature of the mtDNA molecule generates an alignment problem in the region where the start (base pair 1) and the end (base pair 16569) merge [58]. The alignment in this region has less quality and some reads are depreciated due to alignment being made through a lineal molecule. To solve these problems, other authors used tools such as CircularMapper [59] or had the first 500 bp at the end of the reference mtDNA molecule [35]. In the present work, to avoid the circularity of the mtDNA molecule the NCR sequence was linearized, allowing all reads to align over it without the circularity problem. Alignment was performed with bwa aln and samse/sampe [43], as it works better for aDNA than other aligners [60].

For the variant calling, indels, multi-nucleotide polymorphisms (MNPs), and complex events were ignored. When *vcfallelicprimitives* from vcflib [46] is set, MNPs are transformed into single-nucleotide polymorphisms, allowing the pipeline and the rest of the programs as HaploGrep2, to work correctly. Moreover, the region between bases 303 and 315 was excluded due to its alignment complexity. This region involves a PolyC tract that can be interrupted by one T [61, 62]. Additionally, the possibility of both length and point heteroplasmy, makes the automatic analysis more difficult and the presence of damage increases the problems that this region represents. The combination of all these facts generates a challenge to the automatic interpretation of this complex region, so the pipeline avoids this region. However, the analysis of this region can be done manually, by visualizing the alignment with the IGV program [38].

This pipeline has been proven to work for data obtained by PowerSeq^TM^ CRM Nested System kit (Promega Corporation) (coverage: 16013–16569; 1–592). It should work without any modification with the ForenSeq mtDNA Control region kit (Verogen, San Diego, USA) (coverage: 16008–16569; 1–595) and the precision ID mtDNA Control Region Panel (ThernmoFisher Scientific, Waltham, USA) (coverage: 15954–16569;1–610) since the reference sequence employed covered from position 15901 to position 700. The pipeline should be modified for different amplicons from different regions: new reference sequence, new starting coordinates, and removing the coordinates restructuration based on the origin of replication. For non-overlapped amplicons, a major adaptation of the pipeline should be done, and future versions of the pipeline may explore these aspects.

In summary, we confirmed that the better tool to remove duplicates from NGS mtDNA data derived from amplicon-based methods would be fastp v0.23.2 and we established that PMDS = 1 from PMDtools v0.23.2 is the best alternative to analyze ancient samples. To be considered authentic, the sequencing of mtDNA-CR from ancient samples must have a minimum of 9.17% of damaged reads. Furthermore, we developed an easy-to-use pipeline to analyze ancient mtDNA data generated with NGS technology, providing information about possible contamination of the sample and the mtDNA haplogroup and haplotype. However, particularly for ancient samples, some manual analyses may be required to fully validate results since the amplicons that used to be more easily recovered were the ones that had fewer reads with damage, indicating that special care must be taken for poor recovered samples.

## Supporting information

S1 File Supporting information. Summary of deduplication analysis and Post-mortem Molecular Damage (PMD) analysis in genomic samples across different centuries (S1.1 File), mean depth coverage and percentage of damage for the ten analyzed amplicons (S1.2 File), and information about the outputs provided by the script (S1.3 File).
(XLSX)

S1 Fig Screenshots from the IGV program for sample 29. Black frames delimit each stage of the sample: A) with duplicated reads; B) without duplicated reads, removed by fastp software; C) without duplicated reads, removed by MarkDuplicates software. Grey arrows symbolize the aligned reads and coloured rectangles indicate a change of base from the reference sequence (red for Thymine, green for Adenine, blue for Cytosine, and brown for Guanine).
(TIF)
