# Supplementary figures and images for: An easy-to-use pipeline to analyze amplicon-based Next Generation Sequencing results of human mitochondrial DNA from degraded samples
Source: PLoS One. 2024 Nov 21;19(11):e0311115. doi: 10.1371/journal.pone.0311115 (PMC11581256; doi:10.1371/journal.pone.0311115)

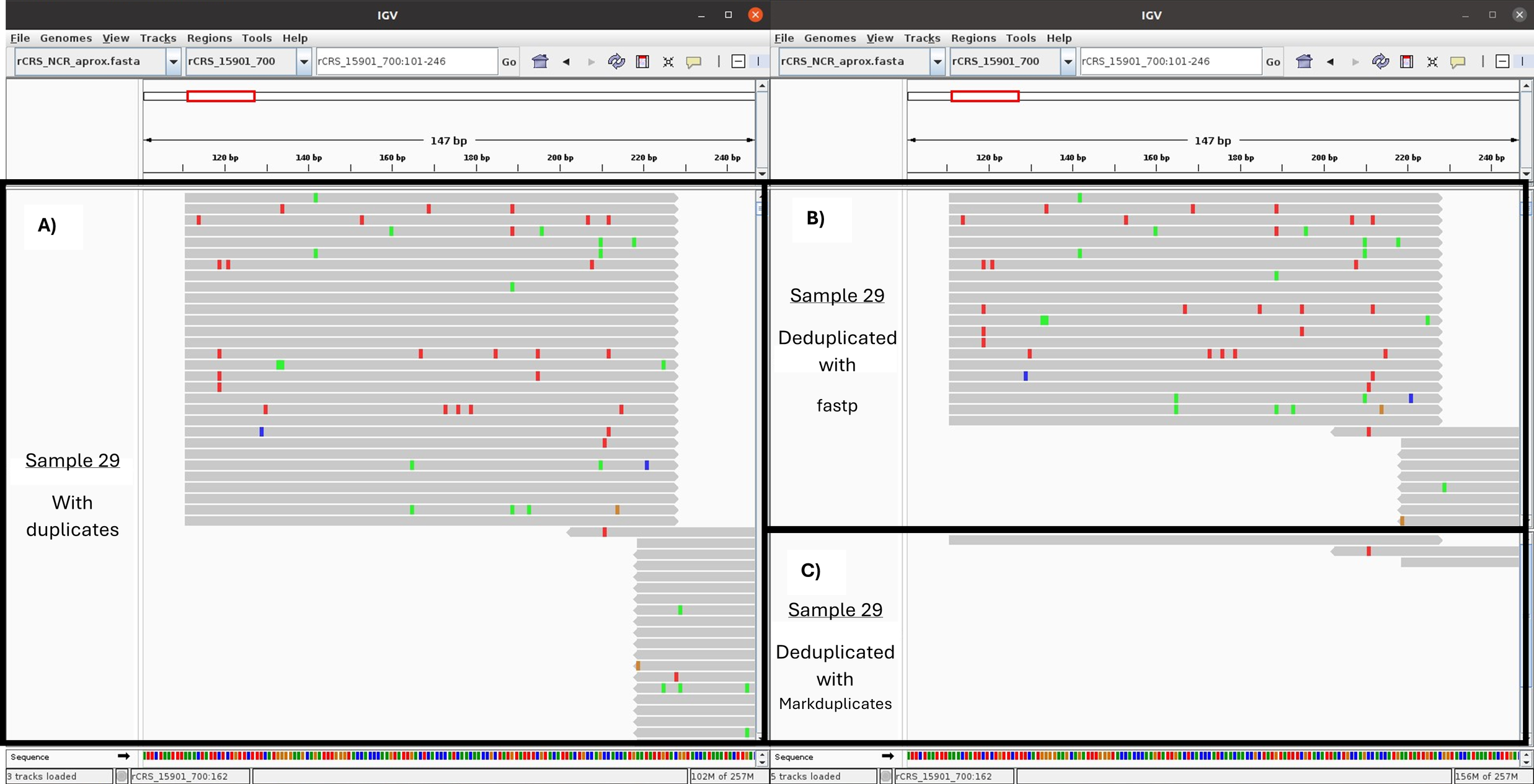

Supplement: S1 Fig — Black frames delimit each stage of the sample: A) with duplicated reads; B) without duplicated reads, removed by fastp software; C) without duplicated reads, removed by MarkDuplicates software. Grey arrows symbolize the aligned reads and coloured rectangles indicate a change of base from the reference sequence (red for Thymine, green for Adenine, blue for Cytosine, and brown for Guanine). (TIF) [file pone.0311115.s002.tif]
